# Aquaculture as a source of empirical evidence for coevolution between CRISPR-Cas and phage

**DOI:** 10.1098/rstb.2018.0100

**Published:** 2019-03-25

**Authors:** Ville Hoikkala, Gabriel M. F. Almeida, Elina Laanto, Lotta-Riina Sundberg

**Affiliations:** Centre of Excellence in Biological Interactions, Department of Biological and Environmental Science and Nanoscience Center, University of Jyvaskyla, PO Box 35, 40014 Jyvaskyla, Finland

**Keywords:** CRISPR, aquaculture, ecology, coevolution, bacteria, phage

## Abstract

So far, studies on the bacterial immune system CRISPR-Cas and its ecological and evolutionary effects have been largely limited to laboratory conditions. While providing crucial information on the constituents of CRISPR-Cas, such studies may overlook fundamental components that affect bacterial immunity in natural habitats. Translating laboratory-derived predictions to nature is not a trivial task, owing partly to the instability of natural communities and difficulties in repeated sampling. To this end, we review how aquaculture, the farming of fishes and other aquatic species, may provide suitable semi-natural laboratories for examining the role of CRISPR-Cas in phage/bacterium coevolution. Existing data from disease surveillance conducted in aquaculture, coupled with growing interest towards phage therapy, may have already resulted in large collections of bacterium and phage isolates. These data, combined with premeditated efforts, can provide empirical evidence on phage–bacterium dynamics such as the bacteriophage adherence to mucus hypothesis, phage life cycles and their relationship with CRISPR-Cas and other immune defences. Typing of CRISPR spacer content in pathogenic bacteria can also provide practical information on diversity and origin of isolates during outbreaks. In addition to providing information of CRISPR functionality and phage–bacterium dynamics, aquaculture systems can significantly impact perspectives on design of phage-based disease treatment at the current era of increasing antibiotic resistance.

This article is part of a discussion meeting issue ‘The ecology and evolution of prokaryotic CRISPR-Cas adaptive immune systems’.

## 1. Introduction

Bacteriophages (phages), the obligate viral parasites of bacteria, pose a constant threat of infection on their hosts. By consequence, a wide range of prokaryotic defence mechanisms have evolved. Phage infection and replication can be blocked in various ways, including preventing phage entry by modifying relevant surface receptors (surface modification, SM) or by degrading an intracellular phage genome using restriction modification (see review [1]). These well-known examples of innate defences, while central to prokaryotic immunity, lack the ability to store and update immunological memories of genetic invaders. Adaptive immunity in prokaryotes is represented by loci known as *c*lustered *r*egularly *i*nter*s*paced *p*alindromic *r*epeats and associated *cas* genes (CRISPR-Cas). CRISPR-Cas loci can be reprogrammed by the acquisition of distinct genetic sequences from invading nucleic acids, thereby preparing them for recognition and termination of upcoming infections with similar sequences [2–5]. CRISPR-Cas research is central to microbiology and biotechnology, but its ecological and evolutionary consequences are still surprisingly poorly understood. While several studies have addressed CRISPR-Cas and phage genomics in natural populations (see below), studies linking genetic and phenotypic data from repeatedly sampled isolates are largely missing [6]. We believe this is partly owing to the generally low number of relatively stable phage–bacterium systems in the environment, which could be repeatedly monitored over longer timescales.

Intensive farming systems are considered as hotspots for the evolution of pathogens, as high densities of susceptible hosts promote pathogen transmission and virulence [7–9]. This feature of the farming environment is also likely to extend to biological interactions between pathogenic bacteria and their phages, owing to increased (pathogenic) bacterial population sizes and phage–bacterium interactions. Aquaculture, the farming of fishes and other aquatic species is one of the fastest growing sectors in food production, providing high-quality protein for human consumption [10]. The enrichment of naturally occurring pathogens in aquaculture environments opens an attractive opportunity to study phage–bacterium coevolution and to observe the functioning of CRISPR-Cas in semi-natural conditions.

Existing national disease surveillance projects for aquaculture-related bacteria and increasing efforts to isolate phages against these pathogens can provide essential empirical information for understanding phage–bacterium coevolution. To estimate whether generalizations derived from simplified laboratory coevolution studies reflect interaction dynamics in natural, semi-natural and clinical settings, more empirical evidence on (i) phage–bacterium interactions at wider taxonomic scale and (ii) different CRISPR-Cas systems are needed. In this review, we discuss the general properties of aquaculture as a domain for phage–bacterium coevolution and examine the position of CRISPR-Cas in these settings. From a practical point of view, it is important to understand the effects of adaptive bacterial immunity for applications such as phage therapy in aquaculture.

## 2. CRISPR-Cas, the adaptive immune system of prokaryotes

CRISPR-Cas operation is divided into two main stages. During the adaptation stage, a protein-complex (comprising of at least Cas1 and Cas2) excises a short sequence from an invasive phage genome. This sequence, known as a spacer, is integrated into the CRISPR array of the host's CRISPR-Cas locus [11]. In the interference phase of CRISPR-Cas, an infection leads to transcription of the CRISPR array. The transcript, called pre-crRNA (CRISPR-RNA), is processed to smaller fragments and used to guide endonucleases, such as Cas9, to corresponding positions in the phage genomes (protospacers). This results in cleavage of invading genetic material and termination of the infection. In addition to phage genomes, CRISPR-Cas systems can target plasmids and other mobile genetic elements (MGEs) and, as such, act as a barrier for horizontal gene transfer [12]. Other roles for CRISPR-Cas have also been reported, including biofilm formation, sporulation, DNA repair and regulation of virulence (see review [13]). Phages may evade CRISPR-Cas by modifying their protospacers [14] or by producing anti-CRISPR proteins [15].

The unique ability of CRISPR-Cas to store genetic information from infections, often in chronological order, opens exciting opportunities for microbial evolution research. Past infections are revealed by the CRISPR arrays, in which the oldest spacers may date back hundreds of thousands of years [16], while the acquisition of novel spacers may be monitored in almost real-time (e.g. [17]). Multiple studies have addressed spacer dynamics in natural populations [18–20] and corresponding changes in phage genomes [6,21,22]. While showing that CRISPR-Cas is active and adaptive in nature, these studies also demonstrate that the diversity of spacer content varies drastically between species, reflecting both the extent of interactions with phages and the relative importance of CRISPR-Cas amidst other defence mechanisms (see review [23]). In addition to acquiring and losing individual spacers, reassortment of CRISPR-Cas loci may be important in shaping spacer profiles in nature [20]. Spacer diversity and corresponding changes in phage protospacers may also be used as a metric for the level of asymmetry in evolutionary potential between bacteria and phages [24]. These dynamics have direct ecological and evolutionary effects in hotspots of pathogen emergence, such as aquaculture settings, but have not been thoroughly investigated with long-term sampling.

## 3. Aquatic and aquaculture environments as domains for phage–bacterium interaction

The complexities of natural habitats contrast the simplified settings of laboratory experiments. Dynamics of bacterial immunity observed *in vitro*, shielded from diverse biotic and abiotic factors, may therefore not be directly translatable to natural environments [24]. To clarify this separation, we review the distinct features of aquatic environments that may contribute to different ecological and evolutionary outcomes in natural settings.

### (a) The ecology of aquatic environments

The probability of phage–bacterium interactions is dictated not only by the abundance, but also by the distribution of bacterial cells and phage particles. Although the numbers of bacteria and their phages in aquatic environments are enormous (e.g. [25]), their distribution is asymmetric. Bacteria congregate largely in biofilms [26,27], but the physical characteristics of water allow transmission of microbes even for long distances. Free-floating (planktonic) cells may drift in currents or actively move towards attractants or from repellents [28]. Furthermore, aquatic microbial communities are composed of several species and the abundance of each varies between microhabitats, also depending on biotic and abiotic factors [29,30].

Phages, on the other hand, have no capacity for active movement, and thus are either drifting randomly by Brownian movement in the water column or associated with organic matter or sediments [29]. A chance encounter between a phage and a bacterium may appear to be a rare event when considering their small sizes and asymmetric distribution, the large volumes of water and the near atomic distances needed for interaction. However, as phage–host interaction is a strong evolutionary driver, it is likely that mechanisms to increase encounter rates have been favoured by evolution. Firstly, and evidently, the high number of phage particles in the environment increase likelihood of encounters with the hosts. Second, a broader host range for the phage (polyvalency) may increase the chance of successful infections. While most phage isolates investigated in laboratory conditions are host-specific, polyvalency has been suggested to be prevalent in natural communities [31]. Third, although phage infections are often considered harmful for bacteria, they may also benefit bacterial populations and promote selection for mechanisms that favour encounters resulting in relationships with mutualistic phages. Phages may contribute to bacterial pathogenicity by providing virulence factors [32], by protecting the bacterium against other phages via superinfection exclusion mechanisms (e.g. [33]), or by restructuring communities through ‘killing the winner’ dynamics [29].

### (b) Aquatic metazoans provide territory for phage–bacterium coevolution

In aquaculture, aquatic animals (metazoans) exist in confined, high-density populations. Metazoans are covered in mucus, which provides a physical and an immunological barrier for the animal. Skin mucosal surfaces are also one of the most nutrient-rich surfaces available for aquatic microbes. By eliciting positive chemotaxis stimuli, they attract both beneficial microbes as well as pathogens, as exemplified by molecular data from European eels [34]. In this species, bacteria selected by mucus were shown to have heightened resistance against host immunity, metals, antibiotics and amoebas. Additionally, these species were abundant in genes related to biofilm formation, bacterial communication and displayed evidence of horizontal gene transfer. Mucosal surfaces may provide a natural habitat for pathogen evolution and emergence, acting as an intermediate niche between water and host that selects microbes best adapted to survive and colonize mucus.

Metazoan mucus layers have also been found to be enriched with phages [35]. This finding has led to the proposal of the bacteriophage adherence to mucus (BAM) model, which predicts an important yet so far overlooked symbiosis between metazoans and phages. Phages are concentrated by weak interactions with mucus components, creating a ubiquitous non-host derived immunity against bacterial invaders during the mucus colonization process [35]. In addition, interaction with mucins leads to subdiffusive motion patterns (in contrast to expected Brownian movement) and promotes phage persistence inside the mucosal layer despite continuous mucus shedding [36]. This would favour phage–bacteria interactions since any bacterial invader, be it pathogenic or not, would end up finding its phage when colonizing the animal. On the other hand, by interacting with mucus components, phages may solve the problem of finding hosts in an open water system by concentrating themselves on the substrate favoured by bacteria.

The enrichment of phages in mucus, coupled with the constant influx of bacteria trying to colonize this environment, makes metazoan mucosal surfaces a hotspot for phage–bacterium interactions. While these interactions can take place in the free water column or in the sediments, it has been suggested that mucus-based encounters are favoured by evolution [36]. The implications of phage–bacterium interaction on metazoan mucosal surfaces may therefore be of great importance to coevolution and to phage therapy, and has been so far overlooked. This is especially important for aquaculture systems, as fish skin and gills are covered with a mucus layer and often targeted by bacterial pathogens. The dynamics of phages and bacteria in the mucus can even be more complex since spatial structuring of mucus has been speculated to have a role in phage replication strategies [37].

### (c) Effects of aquatic niches on bacterial immunity

The environmental heterogeneity of aquatic settings is likely to have consequences for bacterial immunity, as changes in phage and nutrient abundance may select for specific defence strategies. The efficiency of CRISPR-Cas has been predicted to decrease under increasing viral diversity using both theoretical models [38,39] and practical experiments [40]. As diversity correlates with mutation rates and population sizes, higher abundances of phages are predicted to result in immunity mediated by mechanisms other than CRISPR-Cas, such as SM [39]. Simply translating to aquatic settings, water columns with low phage concentration can therefore be predicted to favour CRISPR-Cas, whereas SM would be promoted by phage-rich mucosal surfaces [35]. However, opposing selective forces arise from the fact that SM often compromises the pathogens' ability to colonize their host (see review [41]). Trade-offs associated with SM can therefore also be expected to promote alternative, less costly defences in niches where colonization is prioritized. The resulting defence strategies are further complicated by other abiotic and biotic factors such as migration from the environment and the use of antibiotics (see below). Empirical evidence of alternating defence strategies in aquaculture have been found in the fish pathogen *Flavobacterium columnare*. Its CRISPR-Cas loci are active in these settings [6], but upon exposure to a high titre of phage in laboratory settings the colony morphotype changes to a phage-resistant and non-virulent one [42].

## 4. CRISPR-Cas and phage life cycles

Maybe surprisingly, the majority of lysogens (bacteria carrying temperate phages) also carry CRISPR-Cas systems [43]. Among aquaculture-related bacterial species, at least *Flavobacterium psychrophilum* [44] and *Vibrio anguillarum* [45] carry temperate phages that can be induced into the lytic cycle. However, the interaction between CRISPR-Cas and phage life cycles has remained poorly understood. A central dilemma arises when spacers target an integrated phage genome, as self-targeting is generally lethal. Some prophages overcome this problem by coding for anti-CRISPR proteins that suppress the immune system [15].

The phage–bacteria–metazoan mucus interactions also play a role in lysis–lysogeny switches [37], which may have significant implications for phage–CRISPR-Cas coevolution in aquaculture settings. Lysogeny may be favoured in lower mucus concentrations (outer layer) and lysis in higher concentrations (inner layers). This piggyback the winner (PtW) model would allow bacteria containing the phages to enter the mucosal layer and, when deep enough, undergo a lytic infection and release more phages [37]. Whereas the BAM model [35] can benefit phages by favouring encounters with the hosts, the PtW model [37] may benefit the bacterial hosts by favouring lysogeny. The metazoan that provides the mucosal environment benefits from both by becoming protected from invaders.

During unfavourable conditions for the host, phages can also establish alternative lifestyles such as pseudolysogeny [46]. Here, the phage chromosome is not integrated nor replicated, but inherited by one of the two daughter cells. Pseudolysogeny has been suggested to increase the effective lifespan of phage genomes by keeping it safe from outside host conditions [46]. The role of CRISPR-Cas in initiation and maintenance of this life cycle is unknown. Pseudolysogeny might also be a prevalent life cycle in aquaculture-related phage–bacterium systems, as farming settings are often subjected to seasonal changes that cause variability in phage life cycles and phage–host interactions.

## 5. CRISPR-Cas in relevant aquaculture pathogens

Although there are many potential aquaculture-associated phage–bacterium systems that could elaborate how CRISPR-Cas functions in these environments, such studies are few in numbers. To inspire further research, we compiled the most important sequenced aquaculture pathogens and examined their CRISPR content using publicly available complete genome assemblies and existing publications. We determined CRISPR-Cas types of 24 aquaculture-relevant species. Eleven (approx. 46%) were found to carry a CRISPR-Cas locus in at least one strain (table 1). This analysis reveals that datasets for further CRISPR-phage coevolutionary studies already exist, as phages have been isolated against many of these bacterial species (electronic supplementary material, table S1). Since our analysis was limited to complete genomes, additional CRISPR-Cas systems are likely to arise in species that have not yet been thoroughly sequenced. Below, we highlight two groups of important aquatic pathogens derived from our analysis.

**Table 1.** CRISPR-Cas systems in aquaculture pathogens. (Dark grey cells indicate that a feature (either no CRISPR-Cas or a CRISPR-Cas subtype) is present in all analysed strains of the given species. Light grey cells indicate presence of a feature in some of the strains. The number of strains with the specified feature is displayed in each individual cell and the total number of analysed strains is displayed in parentheses after the species. The table was compiled using CRISPRdisco [47]. Only complete genomes in NCBI's database with CRISPR-Cas loci containing both *cas* genes and CRISPR arrays were considered. Non-pathogenic subspecies were excluded from analysis. Putative CRISPR-Cas systems (type IV and V-U) were excluded from analysis similar to Crawley *et al*. [47]. For details and complete list of genomes see the electronic supplementary material, table S1.)

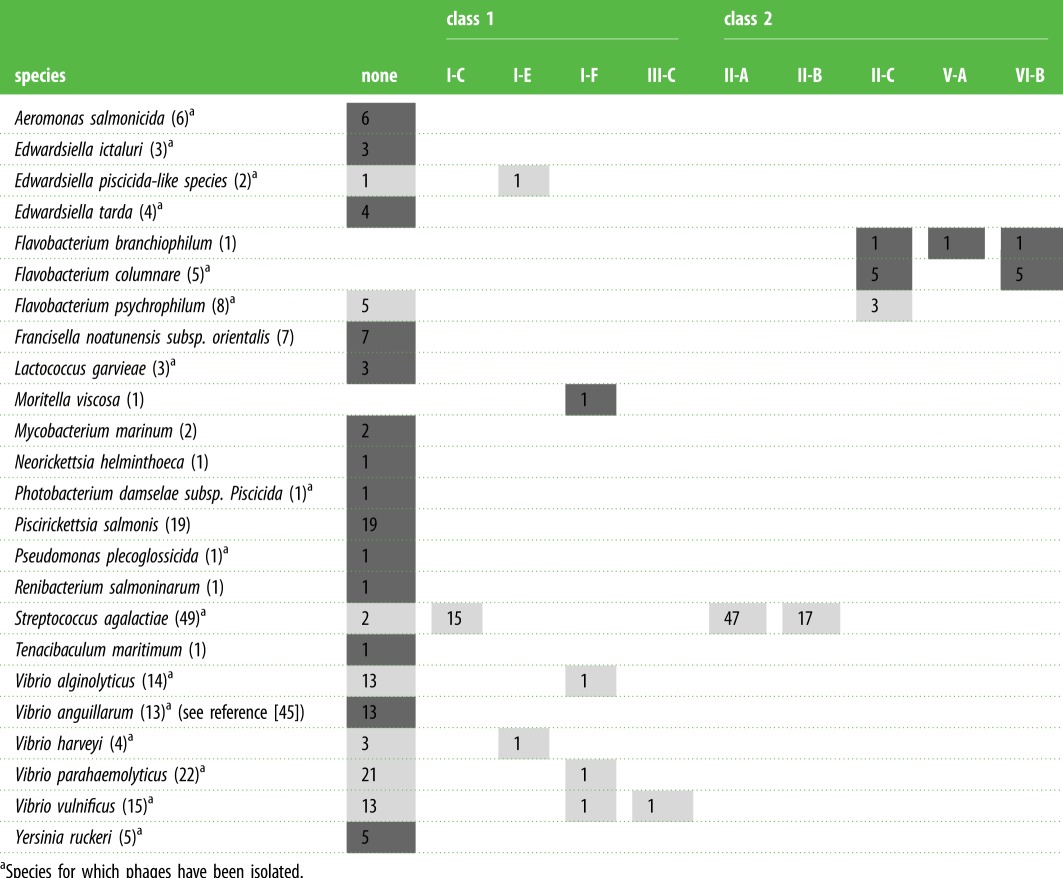

^a^Species for which phages have been isolated.

### (a) *Vibrio* species

*Vibrio* species are abundant in aquatic environments [49,50] and many of these species are associated with diseases of farmed fishes and shrimp, known as vibriosis [51,52]. While most *Vibrio* strains are devoid of CRISPR-Cas, individual strains carrying CRISPR-Cas were found in four out of five *Vibrio* species (table 1). In addition, a recent study on *V. anguillarum* showed that the bacterium shares evolutionary history with a H20-like prophage, and that CRISPR spacers targeting this prophage are widespread across many *Vibrio* species [45]. Another study, using multiple strains of *Vibrio parahaemolyticus*, found positive correlation between the occurrence of virulence factors and CRISPR-Cas elements [53]. *Vibrio parahaemolyticus* and its phages are also the source of the recently discovered anti-CRISPR protein acrF9 [54].

### (b) *Flavobacterium* species

The genus *Flavobacterium* is comprised of 130 species, some of which infect freshwater fishes and cause major economic losses in fish farming across the globe [48]. The most important aquaculture pathogens are *F. psychrophilum, F. columnare* and *Flavobacterium branchiophilum*, which all carry class 2 CRISPR-Cas loci (table 1).

The *F. branchiophilum* genome contains three CRISPR-Cas loci: a type V-A locus associated with the Cas12 (Cpf1) nuclease, a type VI-B locus with the RNA-targeting ribonuclease Cas13b and a type II-C locus with Cas9. Interestingly, *F. columnare* also carries the II-C and VI-B systems and *F. psychrophilum* the II-C system. Recurrence of these class 2 systems in these species may be owing to shared evolutionary history or recent horizontal gene transfer, which is known to promote transmission of CRISPR loci across species [55,56].

Flavobacteria and their phages isolated from fish farms have already contributed to our understanding of phage–bacterium coevolution in semi-natural settings. Repeated long-term (2007–2014) sampling of *F. columnare* and its phages from an aquaculture site revealed temporal dynamics of the CRISPR-phage coevolutionary arms race [6]. Over time, the bacterial host incorporated novel, phage-matching spacers in both type II-C and type VI-B CRISPR-Cas loci. Genome sequencing of the phages revealed cases where the presence of CRISPR spacers in the host population was followed by changes in the corresponding phage protospacer regions, and even subsequent loss of spacers in the host population. However, this study also demonstrated that in addition to CRISPR-Cas, innate resistance mechanisms are also important drivers of genomic and phenotypic evolution in the phage population, which may eventually lead to a broader host range and higher infectivity of the phage.

Studies on the fish pathogen *F. psychrophilum* have demonstrated variance in the number of CRISPR-Cas loci in different strains [57]. Whereas a previous laboratory experiment suggested that CRISPR-Cas may not be active in this species [58], metagenomic samples showing high spacer diversity [59] suggest that CRISPR-Cas defence may be effective under natural conditions. Furthermore, comparison of CRISPR spacers with phage genomes has revealed that especially prophage 6H and its close relatives are ubiquitous companions of *F. psychrophilum,* with a worldwide distribution [57].

## 6. Practical aspects

### (a) CRISPR-Cas in strain typing

CRISPR arrays may reflect previous phage infections, the rejection of plasmids or genetic matter of unknown origin [60]. In some species, spacer content is highly conserved (suggesting a lesser role for CRISPR-Cas in coevolutionary interactions), while in others the spacer profile constitutes a fingerprint that is often unique enough to distinguish otherwise nearly clonal strains of the same species (see review [23]). In fact, spacer-based typing (spoligotyping) was developed long before CRISPR's role as an immune system was uncovered [61] and has played an important role in typing strains of *Mycobacterium tuberculosis* [62]. CRISPR-typing has since been applied to many clinically relevant species, often in combination with other typing-methods [63].

If phage and MGE populations in different aquaculture facilities imprint unique spacer profiles on CRISPR-Cas positive species, the resulting diversity could be used for epidemiological and surveillance purposes. This would require establishing databases of bacterial strains, their respective spacer profiles and isolation sources, and could prove useful in tracking the spread of epidemics and characterizing bacterial diversity during outbreaks. While relevant aquaculture pathogens have not been studied from this point of view, correlations between geographical location and CRISPR arrays have been shown in other bacterial species [64–66].

### (b) CRISPR-Cas and phage therapy

Phage therapy is considered to be an alternative or complement to antibiotic use, and has been used successfully in aquaculture-relevant settings related to mollusc, fish and crustacean diseases [67–69]. Understanding the role of CRISPR-Cas in phage–bacterium interactions may be central to the success of phage therapy aimed towards CRISPR-Cas positive species in aquaculture (table 1). By principle, phage therapy will significantly increase phage–bacterium interaction rates, which will promote evolution of bacterial resistance, both via innate mechanisms and CRISPR-Cas. This will have consequences for the success of phage therapy, but also for leakage of both the phage and the resistant bacterial strains into the environment. Circulation of phage-targeting CRISPR spacers in the environment has been suggested to cause corresponding evolutionary change in the phage population [6].

Maintaining up-to-date CRISPR spacer profiles of bacterial pathogens at fish farms may support phage therapy interventions. As phages are often strain-specific [70,71], they must be chosen carefully to target the prevalent bacterial community. Monitoring spacers and protospacers could also aid in experimental selection or genetic engineering of infective phages. While bacterial resistance is less likely to arise in cases where phage cocktails are used [72], monitoring the emergence of novel spacers may be used in designing new cocktail combinations.

### (c) Use of antibiotics in aquaculture

Fish bacterial diseases are treated with antibiotics, which are usually given in feed. It has been estimated that 30–80% of antibiotics leak into the water owing to excretion and uneaten pellets [73]. While the antibiotic load in fishes is likely to stay at a clinical dose (thus preventing bacterial infections), minor levels of unabsorbed antibiotics are likely to affect phage–bacterium coevolution outside the host. The presence of antibiotics may increase antimicrobial resistance genes carried in the phage genomes [74,75] and contribute to prophage induction [76,77]. Both antibiotics [78,79] and phage infections [71,80] individually increase bacterial mutation rate and fitness. Interestingly, simultaneous exposure of bacteria to antibiotics and phages have been shown to increase resistance to both [81]. However, how exposure to antibiotics influence phage–bacterium coevolution and CRISPR-Cas based resistance outside laboratory conditions has remained less understood. The use of antibiotics in aquaculture and the tendency of CRISPR-Cas to target any incoming MGEs [12] may have important consequences for the spread of antibiotic resistance. Mutants with deprecated CRISPR-Cas systems (or species with no CRISPR-Cas to begin with) may undergo positive selection during antibiotic exposure, thereby increasing the proportion of antibiotic resistant strains with increased phage sensitivity [82–85].

## 7. Conclusion

Aquaculture provides semi-natural and relatively stable habitats for microbial communities, enabling repeated sampling over long time periods. While most aquaculture-related pathogens, as bacteria in general, lack CRISPR-Cas, there are still many tractable species that harbour this immune system (table 1). Individual research groups and national disease surveillance laboratories have undoubtedly already collected numerous pathogenic bacteria and phage isolates from these settings over decades. While only a fraction of the collected phage and bacterial isolates have been sequenced and submitted to databases, it is evident that isolates already exist to conduct studies that can reveal details of phage-CRISPR-Cas dynamics in nature. Coupling isolate-based approaches with metagenomics may be the most effective method for scoping natural bacterial and phage communities, as this would strengthen the (often weak or missing) link between genotype and phenotype. The preferred bacterial resistance mechanisms are likely to vary across planktonic and mucosal environments in accordance with nutrient availability and phage pressure [38–40]. Therefore, models such as BAM [35] and PtW [37] need to be integrated into phage–bacterium interaction studies with fishes, other eukaryotes and mucosal surfaces. Datasets collected from aquaculture environments may also be useful in studying other cellular functions of CRISPR-Cas, such as virulence or biofilm forming capacity.

## Supplementary Material

CRISPR-Cas in aquaculture pathogens

## Data accessibility

This article has no additional data.
